# Preservation of Anatomical Specimens

**Published:** 1871-02

**Authors:** 


### PRESERVATION OF ANATOMICAL SPECIMENS.

Dr. Thos. Dwight, Jr., of Boston, at the last annual meeting of the Massachusetts Medical Society, read a paper detailing some experiments to effect the preservation of dissected anatomical specimens, so that they may retain their original properties indefinitely. Such a consummation he regarded as exceedingly desirable, as it would furnish not only the teacher of anatomy means whereby he could illustrate parts of his subject without actual dissections before his class, but it would provide the surgeon with means of refreshing his memory before an operation, such as neither plates nor dried preparations can give.

He first experimented with a mixture of carbolic acid and glycerin (1 to 5), thoroughly rubbing it into the specimen (a dog's leg). This preserved the form and flexibility of the muscles perfectly, but they were of a dark color. To avoid the discoloration, retaining the preservative effect, he used various combinations of this mixture with alcohol, chloride of sodium, and nitrate of potash. It was found that great care is needed, when using the alcohol, to avoid getting so much as to harden the tissues and to prevent their flexibility; and with too little alcohol and omission of the carbolic acid, a dipocere was developed after a few weeks; this, however, he was able to check by bathing the part in alcohol.

His conclusion is, after many experiments, that for soft, muscular structures, the following mixture is superior to all others: To a mixture of 6 parts glycerin to 1 part alcohol, there is added in excess a mixture of chloride sodium 2 parts, and nitrate potassa 1 part.

This is thoroughly rubbed into the specimen, which is then rolled in a cloth wet with the mixture. In the course of a week or two the part is again rubbed with the preservative material. In a fore-arm dissected to show muscles, arteries, and nerves, preserved with this mixture, he says, "the color, though not red, is better than in the preceding specimens, and in all other respects it is almost perfect."

For preserving specimens of bone and cartilage, with membranes and ligaments attached, and for hard tumors and the like, he prefers a mixture of glycerin 7 parts, alcohol 3 parts, and carbolic acid 3 parts. This he injects into the medullary cavity of bone and rubs thoroughly into the outer surface.

In preserving soft specimens, the blood must be entirely removed by soaking or injecting water through the vessels; when the latter is resorted to, a little carbolic added to the injection is beneficial.

Any drying subsequent to the preserving process is easily prevented by applying a little glycerin.

